# Occurrence of Potentially Toxic Metals Detected in Milk and Dairy Products in Türkiye: An Assessment in Terms of Human Exposure and Health Risks

**DOI:** 10.3390/foods14152561

**Published:** 2025-07-22

**Authors:** Burhan Basaran

**Affiliations:** Department of Nutrition and Dietetics, Faculty of Health Sciences, Recep Tayyip Erdogan University, 53100 Rize, Türkiye; burhan.basaran@erdogan.edu.tr

**Keywords:** dairy products, kefir, children’s milk, metal exposure, risk assessment

## Abstract

This study investigated ten potential toxic metals (PTMs) in six milk and dairy product types and evaluated food safety (TDI, RDA), human exposure (EDI), non-carcinogenic risk (THQ, HI), and contamination levels (CF, PLI). Based on total PTM load, products ranked as: children’s milk > yogurt > protein milk > milk > ayran > kefir. Aluminum (Al) showed the highest average concentration in all products except ayran, where manganese (Mn) was dominant. Cadmium (Cd), mercury (Hg), and lead (Pb) were consistently at the lowest levels. Except for chromium (Cr) exposure from children’s milk, all average and maximum EDI values stayed below TDI and RDA thresholds. Children’s milk had the highest non-carcinogenic risk, while yogurt, kefir, milk, and ayran may also pose potential risks when maximum HI values are considered. Although CF values varied across products, PLI results showed all products had high levels of PTM contamination. Given the widespread consumption of dairy across all age groups, especially by sensitive populations like children, monitoring and controlling PTM levels is crucial alongside ensuring nutritional quality.

## 1. Introduction

Milk and dairy products are among the main sources of many vital nutrients such as protein, calcium, phosphorus, vitamin B_12_, and vitamin B_2_ in human nutrition [1,2]. It is indispensable for protecting bone health and supporting growth and development, especially in childhood, adolescence, pregnancy, and old age [3,4]. Thanks to its high bioavailability of calcium, it contributes to the prevention of bone diseases such as osteoporosis [5]. In addition, the proteins found in dairy products are important in terms of repairing muscle tissues and strengthening the immune system [6,7]. However, studies conducted in recent years have brought to the agenda the risk of contamination with various contaminants in addition to the nutritional value of milk and dairy products. These products can be contaminated with potentially toxic metals (PTMs) during the process from production to consumption, and the accumulation of PTMs can lead to serious health problems in the long term. This study focuses on aluminum (Al), chromium (Cr), manganese (Mn), cobalt (Co), nickel (Ni), copper (Cu), arsenic (As), cadmium (Cd), mercury (Hg), and lead (Pb), which have direct and potential health risks for humans.

The adverse effects of PTMs on human health become more evident, especially in cases of long-term and high-level exposure [8]. PTMs can accumulate in the body and permanently affect many physiological systems, especially the nervous system [9,10]. Al, one of the prominent PTMs in terms of nervous system toxicity, has been associated with neurodegenerative diseases, especially Alzheimer’s disease [11]. Similarly, Mn exposure can lead to a Parkinson’s-like neurological disease called “manganism” [12]. PTMs such as Pb and Hg can negatively affect the development of the central nervous system, especially in children, leading to learning disabilities, cognitive impairment, and behavioral disorders. In addition, Pb can cause permanent neurological damage at an early age, and Hg can cause toxic accumulations in the kidney, liver, and immune system in addition to the nervous system [13,14,15,16]. As and Cd play a role in the development of various organ cancers, especially lung, bladder, and prostate [17,18,19]. Cr has been associated with kidney dysfunction and skin problems [20,21]. Although Cu is an essential element for the body, it can trigger cell death by increasing oxidative stress in case of excessive exposure [22,23]. Similarly, PTMs such as Co and Ni can lead to wide-ranging health problems such as immune system reactions, respiratory tract irritations, and genetic damage [24,25,26]. These findings draw attention to the insidious effects of PTMs, which develop not only through acute but also chronic exposure, and suggest that low doses, especially through the food chain, may increase the risk of serious diseases over time. Therefore, it is of great importance to continuously monitor PTMs levels in foods, especially milk and dairy products, and to keep them within safe limits for public health [27,28,29].

Milk and dairy products are widely consumed as a basic food source for all age groups and have an important place in the food chain. The high nutritional value of these products makes them indispensable, especially for sensitive groups such as children, pregnant women, and the elderly, while at the same time making them foods that should be carefully monitored in terms of possible contaminants. Factors such as chemicals used in agricultural production, animal feeds, industrial activities, and environmental pollution can lead to the accumulation of PTMs in the environment where dairy animals live, and these PTMs can indirectly pass into milk [30,31,32]. Therefore, regular and scientific research on the presence of PTMs in milk and dairy products is of great importance both in terms of ensuring food safety and protecting public health. This study aims to comparatively evaluate the PTMs contents in different milk and dairy products (milk, protein milk, children’s milk, yogurt, kefir, and ayran) and discuss potential health risks.

## 2. Materials and Methods

### 2.1. Materials

This study included a total of 66 products with different brands and contents. These products were classified into six groups as packaged milk (n = 19), protein milk (n = 6), children’s milk (n = 13), yogurt (n = 13), kefir (n = 7), and ayran (Traditional product) (n = 8). The number of samples included in the study was determined by considering their representation levels in the market. The products included in the study were selected from brands offered to consumers in national and local chain stores, supermarkets, and online sales platforms operating in Türkiye and preferred by a wide range of users in the market. Two samples from each product group with different production and batch numbers were provided in their original packaging, and the samples were stored in the refrigerator until analyzed. Some information about the milk and dairy products included in the study is shown in Table S1.

### 2.2. ICP-MS Analysis

#### 2.2.1. Reagents and Standards

All chemicals and reagents used in this study were of analytical reagent grade. Nitric acid (65% HNO_3_), hydrochloric acid (30% HCl), and hydrogen peroxide (35% H_2_O_2_) were obtained from Supelco (Darmstadt, Germany) and Merck (Darmstadt, Germany). The Hg standard solution was purchased from AccuStandard (New Haven, CT, USA), while the ICP multi-element standard solution III, tuning solution, and internal standard solution were supplied by Agilent Technologies (Santa Clara, CA, USA).

#### 2.2.2. Equipments

The analyses were carried out using an ICP-MS 7800 instrument (Agilent Technologies, Tokyo, JHS, Japan). In addition to this, different varieties of laboratory equipment were utilized during sample preparation and analysis, including a microwave digestion system (Milestone Ethos Up, Sorisole, Italy), an ultrasonic cleaner (Shenzhen, Guangdong, China), and an ultrapure water system (Millipore Direct Q 8UV, Billerica, MA, USA).

#### 2.2.3. Sample Preparation and Analysis

The analysis was conducted with certain modifications based on the method described by Basaran and Sadighara [33]. Sample preparation was carried out using a microwave digestion system. For milk and dairy products, the “Bovine milk” protocol provided by the manufacturer was applied. One mL of liquid samples was transferred into teflon digestion vessels, followed by the addition of 9 mL of nitric acid and 1 mL of hydrogen peroxide to establish a closed digestion environment. The samples were then incubated at 210 °C for 15 and 20 min, respectively. After the samples cooled to room temperature, they were transferred to 15 mL falcon tubes and diluted with 15 mL ultrapure water. The resulting solutions were filtered through a 0.45 µm membrane filter and directly analyzed without further dilution using an ICP-MS 7800 system. A blank solution composed of 9 mL HNO_3_ and 1 mL H_2_O_2_ was used for quality control purposes. All analyses were performed in triplicate. Quantitative measurements were automatically processed using the Mass Hunter 4.4 Workstation Software 7800 ICP-MS Top C.01.04. Cd, Hg, and Pb were quantified in standard mode (no gas), while the KED mode was applied for Al, Cr, Mn, Co, Ni, Cu, and As using Helium as reaction gas (Argon). The analytical operating conditions are summarized in Table S2.

#### 2.2.4. Quality Control

Calibration curves were constructed using a series of standard solutions prepared at different concentrations from certified stock solutions. Method performance was assessed through recovery experiments by spiking samples 8, 43, and 62 with 100 µg/L of the target analytes. For each element analyzed, the limit of detection (LOD), limit of quantification (LOQ), calibration curves (R^2^), (concentration range: 0, 10, 25, 50, 100, 250, and 500 µg/L for Al, Cr, Mn, Co, Ni, Cu, As, Cd, and Pb; 0, 2.5, 5, 7.5, 10, and 12.5 µg/L for Hg) and recovery rates are presented in Table S3.

### 2.3. Health Risk Assessment

In this study, a comprehensive health risk assessment was conducted to evaluate the potential adverse effects associated with the consumption of milk and dairy products contaminated with PTMs. The assessment was based on key risk indicators, including Estimated Daily Intake (EDI) and non-carcinogenic risk (Target Hazard Quotient (THQ) and Hazard Index (HI)) (Table 1).

### 2.4. PTMs Contamination Level

The Contamination Factor (CF) serves as a quantitative indicator for assessing the degree of PTMs contamination in food matrices, particularly in multi-metal exposure scenarios. This metric enables comparative risk assessments across different PTMs and samples, supporting the identification of food safety concerns. The CF is derived using the following Equation (1).

CF = C_m_/C_o_ (1)

where Cm represents the analytically determined concentration (µg/kg) of a specific PTM in each sample matrix, and Co is the background level of the PTM. In the absence of standardized baseline levels for heavy metals in milk and dairy products, Salazar-Rojas et al. recommend employing the arithmetic mean of the two lowest concentration values detected among the analyzed samples for each individual PTM as the surrogate Co value [39]. Based on the classification by Sigamani et al. [40], CF is interpreted as follows (Table 2).

The Pollution Load Index (PLI) is employed as an integrative metric to assess the overall degree of sample contamination by PTMs. The PLI is derived using the following Equation (2).

PLI = (CF_1_ × CF_2_ × …… × CF_n_)^1/n^ (2)

where n denotes the number of PTMs assessed, and CF represents the individual pollution index for each PTM. Based on the classification by Sigamani et al. [40], PLI is interpreted as follows (Table 2).

### 2.5. Statistical Analysis

The data obtained in the study were analyzed using IBM SPSS Statistics version 26. The assumption of normality was assessed with the Kolmogorov-Smirnov test. For comparisons between independent groups, Kruskal-Wallis analysis was employed. Post-hoc comparisons were conducted using the Bonferroni test to identify statistically significant differences among groups. Different letters within the same group indicate statistically significant differences (*p* < 0.05). Spearman’s rho correlation coefficient was used to determine the strength and direction of non-causal relationships between two numerical variables. In Spearman’s rho Correlation Coefficient (r) calculations, the coefficients range from −1 to 1. r = 0.00 (no correlation), r = 0.01–0.29 (low correlation), r = 0.30–0.69 (medium correlation), r = 0.70–0.99 (high correlation), r = 1.00 (full correlation).

## 3. Results and Discussion

### 3.1. PTMs Levels in Milk and Dairy Products

In the study, a total of six different milk and dairy product groups were analyzed, and the analysis results were evaluated with some safety limits and in comparison with the existing literature (Table 3). In addition, the findings were evaluated statistically on the basis of PTM (Table S4).

The mean Al concentrations were highest in yogurt (946 μg/kg) and children’s milk (895 μg/L). The lowest Al levels were found in kefir (32 μg/L) and ayran (30 μg/L). Yogurt had higher Al levels than milk and kefir (*p* < 0.05). The Al levels achieved for milk and yogurt in this study were higher compared to other studies conducted in France, Spain, and China [47,50,51].

According to mean Cr levels, the products are listed as children’s milk > yoghurt > protein milk > ayran > kefir > milk. The Cr level of children’s milk and yogurt is higher than milk and ayran (*p* < 0.05). The Cr levels achieved for milk and yogurt in this study are lower compared to other studies [30,47,50,51]. The Cr level detected in milk in the study conducted in the Republic of Korea is more than 70 times higher than this study [52]. The Cr level reported in yogurt in the Slovak Republic is more than 2 times higher than the values achieved in this study [48].

The mean Mn levels were found to be particularly high in children’s milk (300 μg/L) and yogurt (155 μg/kg) groups. Kefir and ayran showed low concentrations of Mn. The Mn levels of children’s milk and yogurt were higher than milk and ayran (*p* < 0.05). The Mn level detected in milk was considerably lower compared to the study of Khan et al. [52]. The Mn level detected in yogurt was consistent with the study of Capcarova et al. [48] and higher compared to other studies conducted in France and the Republic of Korea [50,52].

The highest and lowest average Co levels were detected in children’s milk and milk = kefir, respectively. The Co levels of protein milk, children’s milk, and yogurt were higher than kefir milk and ayran (*p* < 0.05). While the Co level achieved for milk is consistent with the study of Chekri et al. [47], it is generally lower compared to other studies [44,51,52]. In this context, the Co level reported for milk, especially in Brazil [44], is quite remarkable (1050 μg/L). The Co level detected in yogurt is higher compared to other studies [50,52].

In terms of mean Ni concentrations, children’s milk (88 μg/L) and yoghurt (72 μg/kg) products stand out. In contrast, lower levels were recorded in kefir (14 μg/L) and milk (12 μg/L) products. Ni levels in children’s milk and protein milk were higher than in other dairy products, while Ni levels in milk, protein milk, and ayran were higher than kefir (*p* < 0.05). While the Ni level detected in milk was lower compared to other studies, the Ni concentration in yogurt was higher than that reported by Luis et al. [50], but lower than that reported by Capcarova et al. [48].

According to mean Cu levels, products are listed as children’s milk > yoghurt > protein milk > ayran > kefir = milk. Children’s milk and yogurt have higher Cu levels than milk, kefir, and ayran (*p* < 0.05). While the Cu level detected in milk is generally quite low compared to similar studies in the literature, the Cu level in children’s milk is consistent with Pipoyan et al. [43]. The Cu level detected in yogurt is quite high compared to Capcarova et al. [48] and lower than the study by Shahbazi et al. [49]. The Cu level reported for ayran by Hassan and Elarnaoutti [42] is more than 2 times higher than this study.

The mean As level was observed in low concentrations in children’s milk, protein milk, milk, and kefir products. A significant increase was observed in the yogurt product group. The As level of yogurt was higher than milk (*p* < 0.05). The As level detected in milk and yogurt in this study was remarkably high compared to other studies [47,52].

While the mean Cd level in kefir and milk was <LOD, higher Cd levels were determined in children’s milk and protein milk compared to other dairy products (*p* < 0.05). The Cd levels detected in the samples examined in this study were significantly lower than the critical limit (10 µg/kg) determined for Cd in milk [53], and the samples examined were evaluated as reliable. While the Cd levels detected in milk and yogurt samples were significantly lower compared to other studies in the literature, the values detected in kefir were consistent with the study of Sujka et al. [46]. High levels of Cd were reported in studies conducted in Pakistan, Lebanon, and Libya [42].

Hg concentrations were at very low levels (<LOD–0.10 μg/L–kg) in all product groups (*p* > 0.05). Although Codex Alimentarius has set a limit value for Hg in milk, the value it sets for Hg in general foods is 1 μg/kg [53]. Therefore, all samples examined in this study are lower than the critical limit and are reliable. The high level of Hg reported in milk by Sant’Ana et al. [44] is quite concerning.

In terms of mean Pb levels, generally low concentrations were recorded in the products, and the highest Pb value was detected in the ayran group (*p* > 0.05). The Pb levels detected in the milk samples examined in this study were lower than the critical limit determined for Pb in milk (20 µg/kg) [54], and the examined samples were accepted as reliable. Although no statistical comparison was conducted, the Pb levels identified in this study were generally lower than those reported in previous studies [30,42,44,45,46,47].

**Figure 1:**
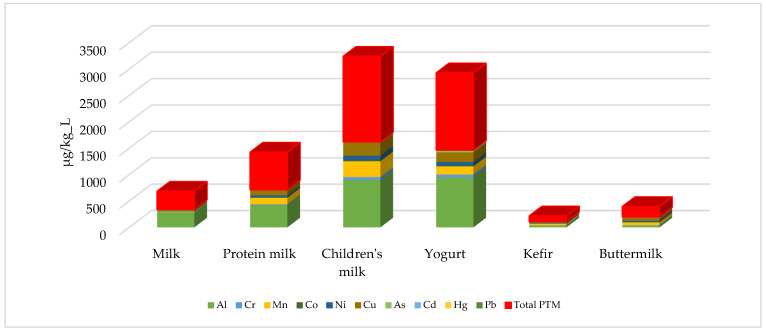
Total PTM load of milk and dairy products.

The results regarding PTM content show significant differences according to product groups. In particular, children’s milk and yogurt products have a high-risk profile in terms of total PTM load according to total PTM load (Figure 1). Potential sources of this situation may include nutrient enrichment processes, additives, and process equipment. High PTM content in products aimed at children, in particular, is a serious concern because children’s metabolic processes absorb PTMs at a higher rate, and their elimination is more limited. Kefir and milk products stand out as safer consumption options with their low PTM content. In light of these results, process optimizations are recommended to reduce PTM contamination in dairy product production. In addition, regular monitoring of PTM concentrations and supporting them with legal regulations are of critical importance in terms of protecting consumer health.

In this study, the levels of certain PTMs detected in milk and dairy products were found to be higher compared to those reported in previous studies. This discrepancy may be attributed to several factors. One primary reason could be the influence of regional environmental pollution on the raw materials and feed sources used in dairy production. Livestock raised near industrial zones are more likely to be exposed to PTMs through contaminated soil, water, and feed. In addition, the production technologies, processing equipment, and storage conditions employed during manufacturing may also contribute to PTM contamination. Therefore, the elevated PTM concentrations observed in this study should be interpreted not only in the context of environmental factors but also in relation to possible variations in production processes and technological infrastructure.

Contamination of PTMs into milk and dairy products is a complex and multi-stage process extending from farm to table. The first and most common source of contamination is animal feed and feed raw materials. This risk increases especially in regions where feed crops grown in soils exposed to PTM contamination or irrigation waters contaminated with industrial wastes are used [55,56]. PTMs such as Cd, Pb, As, and Ni in the soil can pass into plants, which can be consumed by animals and transferred to the systemic circulation and thus to milk. In addition, drinking water sources for animals are an important contamination route; the PTM load of water can show significant differences, especially in urban or industrial areas [57]. The milk processing process constitutes a separate contamination risk area. Stainless steel equipment (especially in 304 and 316 grade steels) can lead to the migration of Cr and Ni ions during processing [55]. In addition, Al alloy containers and piping systems can cause Al migration during processing [58]. In addition, corrosive chemicals used during cleaning and disinfection processes can trigger the release of PTM ions from equipment surfaces [59]. This risk may increase even more in fermented dairy products because fermentation time, microbial acidification, and pH changes can increase the solubility of PTM ions and facilitate contamination [60]. In addition, additives (flavors, stabilizers, vitamin-mineral premixes) and culture starters used for fermentation can also indirectly contribute to PTM exposure [55]. Especially in children’s milks and fortified products, the purity level of minerals intentionally added during formulation is of critical importance; insufficiently purified premixes can lead to unwanted PTM contamination [61]. Finally, packaging materials are another source of risk. PTM migration has been reported through glass bottles with metal caps, cardboard packaging containing laminates, and even dyes and filling agents used in polymer-based containers [57]. It has been shown that PTMs, especially As and Pb, can migrate into the product through polymer migration.

### 3.2. Relationship Between PTMs

**Figure 2:**
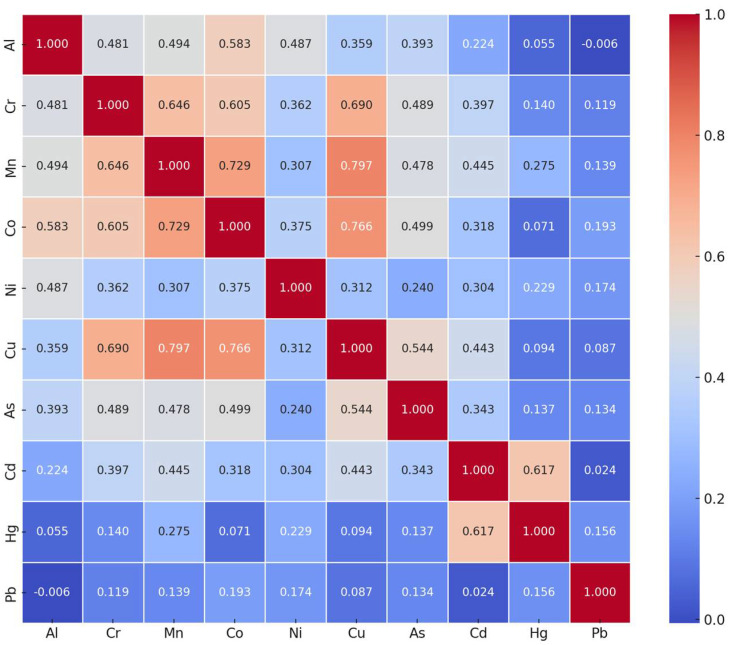
Level of relationship between PTMs.

PTMs are found in nature or in foods, either individually or together. The coexistence of different PTMs strengthens their toxic effects through synergistic interactions between them; this further increases the risks posed by PTMs, especially those ingested through food, on human health. The relationship between PTMs detected in all milk and dairy products in this study is shown in Figure 2.

A moderate positive significant relationship was found between Al and Cr (*p* < 0.01), Mn (*p* < 0.001), Co (*p* < 0.01), Ni (*p* < 0.01), Cu (*p* < 0.05), and As (*p* < 0.01); a moderate positive significant relationship was found between Cr and Mn (*p* < 0.001), Co (*p* < 0.001), Ni (*p* < 0.05), Cu (*p* < 0.001), As (*p* < 0.01) and Cd (*p* < 0.01); a highly positive relationship was found between Mn and Co (*p* < 0.001) and Cu (*p* < 0.001); a moderate positive significant relationship was found between Mn and Ni (*p* < 0.05), As (*p* < 0.01) and Cd (*p* < 0.001); a highly positive relationship was found between Co and Cu (*p* < 0.001). It was observed that there was a moderate positive significant relationship between Co and Ni (*p* < 0.01), As (*p* < 0.001) and Cd (*p* < 0.05), a moderate positive significant relationship between Ni and Cu (*p* < 0.05) and Cd (*p* < 0.05), a moderate positive significant relationship between Cu and As (*p* < 0.001) and Cd (*p* < 0.01), a moderate positive significant relationship between As and Cd (*p* < 0.05), and a moderate positive significant relationship between Cd and Hg (*p* < 0.001). These data provide information about PTMs’ behaviors and provide important clues, especially for monitoring PTMs showing common behavioral patterns.

The number of studies examining the correlation between PTMs detected in milk and dairy products is limited in the literature. Capcarova et al. reported that there was no strong correlation between PTMs detected in yogurt [48]. Sant’Ana et al. reported that there was a moderate negative significant relationship between Cu and Fe, Cu and Zn concentrations, a moderate significant relationship between Pb and Ca, positive correlations between Fe and Se and Ni and Fe, although they were not statistically significant [44]. A high significant positive linear relationship was found between Cd and Pb in processed and raw dairy products, a moderate positive significant relationship between Cr and Pb, and a low positive significant linear relationship between Cr and Cd [30].

### 3.3. Dietary Exposure

In this study, PTM exposure resulting from the consumption of milk and dairy products was calculated and evaluated for different age groups and special populations (Table 4).

The mean Al exposure levels were significantly high, especially in children’s milk (10.2 µg/kg/day). Relatively high levels of Al exposure (1.20–1.73 µg/kg/day) were also determined in yoghurt and protein milks, while very low levels (0.08–0.11 µg/kg/day) were calculated in kefir and ayran products. The tolerable daily intake (TDI) for Al was determined as 286 µg/kg/day [29]. The mean Al exposure from children’s milk consumption contributed approximately 3.6% (max: 13.1%) to the TDI, while the average contribution levels of other dairy products were lower than 1% (max: 1.5%). Although Al is a common element in nature, it is not essential for the human body and can cause adverse effects on human health, especially on children’s health, in case of high-level exposure. Al can have a toxic effect on the nervous system, causing neurodevelopmental disorders, learning disabilities, and behavioral problems [62]. Children with underdeveloped kidney function are at higher risk because they have difficulty excreting Al from the body [63]. In addition, some studies suggest that long-term Al exposure may be associated with neurodegenerative diseases such as Alzheimer’s disease [64,65].

The mean Cr exposure resulting from the consumption of milk and dairy products is generally low. However, the average Cr exposure resulting from the consumption of child milk is 0.59 µg/kg/day, which is higher than other product groups. The Recommended Dietary Allowance (RDA) value for Cr in male and female individuals and children between the ages of 4–8 has been determined as 35, 25, and 15 µg/day, respectively [66]. Accordingly, the contribution rate of the mean daily Cr exposure level (10.3 µg/day) resulting from consumption of children’s milk to the RDA is 69% (max: 228%). The contribution rate (min–max) of the mean Cr exposure level resulting from consumption of milk, protein milk, yogurt, kefir, and ayran to the RDA was calculated as 0–5.73, 0–57.3, 2–34.4, 0–26, and 0–20%, respectively. The contribution rate of the mean total Cr exposure level resulting from the consumption of milk and dairy products to the RDA is 24.5–34.4 (max: 86%). Therefore, the values indicate that children’s milk should be carefully monitored in terms of potential health risks. Chronic Cr, especially its hexavalent form (Cr(VI)), poses a serious threat to human health due to its toxic and carcinogenic effects. Cr(VI) easily enters the cell and triggers oxidative stress, DNA damage, genetic mutations, and cellular death mechanisms [67]. These processes have been associated with the development of various types of cancer, especially lung cancer [68]. Chronic Cr exposure can cause respiratory system disorders (asthma, bronchitis, nasal septum ulceration), skin dermatitis, ulcers, and allergic reactions [20,69]. Children are much more vulnerable to the harmful effects of Cr due to their developing organ systems and higher respiratory and metabolic rates [70].

The mean Mn exposure level (3.37 µg/kg/day) resulting from children’s milk consumption is much higher compared to other products, such as milk and yogurt. The RDA value for Mn in male and female individuals and children aged 4–8 years was determined as 2300, 1800, and 1500 µg/day, respectively [66]. The average Mn exposure level (59 µg/day) resulting from child milk consumption contributes 3.93% (max: 9.7%) to the RDA. The contribution level of Mn exposure resulting from the consumption of each dairy product, including the mean daily total Mn exposure level, to the RDA is <1.5% (max: 6.5%). Therefore, the values indicate that the products are safe in terms of Mn exposure. Although Mn is an essential element for the body, it shows toxic effects in case of high levels of exposure. High Mn exposure can lead to neurotoxicity, causing impairments in memory, attention, and motor skills [71]. In children, effects such as learning difficulties, behavioral problems, and developmental delays can be observed [72]. In addition, Mn accumulation can lead to Parkinson’s-like neurological syndromes and deterioration in liver and kidney functions [12,73].

While the highest Co exposure resulting from consumption of milk and dairy products was detected in children’s milk (0.20 µg/kg/day), exposure values were low in other products (<0.00–0.02 µg/kg/day). The TDI for Co was determined as 8.6 µg/kg/day [74]. The mean contribution level of Co exposure resulting from children’s milk consumption to the TDI was approximately 2.3% (max: 12.7%), while the mean contribution levels of other dairy products were lower than 0.5% (max: 1%). Co is necessary in trace amounts for human health since it is included in the structure of vitamin B12 [75]. However, excessive intake may cause toxic effects. High-dose Co exposure can have adverse effects on the cardiovascular system and cause conditions such as heart muscle enlargement (cardiomyopathy), thyroid dysfunction, and lung damage [25,76,77]. Co can also cause allergic reactions and skin sensitization in some individuals [78].

According to the mean Ni exposure level, the product groups are listed as children milk > yogurt > protein milk > ayran > milk > kefir. It is particularly striking that the mean Ni exposure level (0.86 µg/kg/day) resulting from children’s milk consumption is quite high compared to other product groups. The TDI for Ni was determined as 13 µg/kg/day [79]. The contribution rate of the mean Ni exposure level resulting from children’s milk consumption to the TDI was calculated as 6.6% (max: 16%). The contribution level of the daily mean total Ni exposure level to the TDI was calculated as 1.5% (max: 3%). Ni is a metal widely used in industrial activities and has no physiological function for the human body. Ni is considered safe for most adults in amounts not exceeding 1 mg per day [80]. However, it has been stated that it can cause significant health risks to human health, especially allergic skin reactions, when taken in high doses [24,81,82]. The International Agency for Research on Cancer has defined Ni as possibly carcinogenic to humans in Group 2B [83].

The mean Cu exposure resulting from consumption of children’s milk (2.89 µg/kg/day), yogurt (0.30 µg/kg/day), and protein milk (0.28 µg/kg/day) is remarkably high compared to other foods. The RDA values for Cu in adults (male/female) individuals and children aged 4–8 years were determined as 900 and 440 µg/day, respectively [66]. The mean Cu exposure level resulting from children’s milk consumption (51 µg/day) contributes 11.6% (max: 29.5%) to the RDA. The contribution rate of the mean Cu exposure level resulting from consumption of milk, protein milk, yogurt, kefir, and ayran to the RDA was calculated as 0.08 (max: 0.16%), 2 (max: 9%), 2 (max: 3.5%), 0.1 (max: 5%) and 0.4 (max: 2%), respectively. The contribution level of the mean daily total Cu exposure level to the RDA was calculated as 3.6% (max 11%). Therefore, the values show that the products are safe in terms of Cu exposure. Cu is an essential microelement for the human body and must be taken regularly through water and food [22,84]. Cu is especially important for children’s growth, immunity, iron metabolism, and nervous system development [74,85,86]. However, high levels of Cu exposure can cause oxidative stress, cell damage, and neurological problems in some individuals [23,87,88].

According to mean As exposure levels, the product groups are listed as yogurt > children’s milk > ayran > milk > kefir. Pb exposure level for all products was calculated as <0.00 µg/kg/day. Since TDI values for As and Pb have not been determined, the contribution rates of these metals were not calculated. Although the As and Pb exposure levels arising from the consumption of milk and dairy products are low compared to other PTMs, the potential health risks of As and Pb are important in terms of long-term exposure. As and Pb are two heavy metals that have serious toxic effects on human health. As reduces the resistance to infections by weakening the immune system, causes permanent damage to the nervous and cardiovascular systems, and increases the risk of developing various types of cancer, especially lung, skin, and bladder cancers [89,90]. Pb, on the other hand, poses a great risk, especially for children. It has been reported that it can disrupt the development of the nervous system and cause learning difficulties, attention deficit, and behavioral disorders [57,91,92]. Since both metals show more severe effects in individuals of developing age, limiting exposure is of critical importance for public health. The International Agency for Research on Cancer (IARC) has accepted arsenic and arsenic compounds as directly carcinogenic to humans (Group 1), while metallic lead has been evaluated as Group 2B (Possibly carcinogenic to humans), inorganic lead compounds as Group 2A (Probably carcinogenic to humans) and organic lead compounds as Group 3 (Not classifiable as to its carcinogenicity to humans) [83].

All Cd and Hg exposure levels were calculated as <0.00 µg/kg/day in all product groups. TDI values for Cd and Hg intake were determined as 1 and 0.57 µg/kg/day, respectively [29]. The contribution rates of the mean and maximum Cd and Hg exposure levels calculated for all products to the TDI are quite low. Considering the high toxicity potential of these metals, these low levels are a positive indicator. However, it should not be forgotten that both metals can have adverse effects on human health at low doses. Cd and Hg are heavy metals that have extremely toxic effects on human health. Cd can cause chronic renal failure by specifically targeting the kidneys, and long-term exposure is also associated with respiratory system diseases and some types of cancer [93,94]. Hg has particularly destructive effects on the central nervous system; the methylmercury form can disrupt brain development, leading to motor skill loss, learning disabilities, and cognitive retardation in children [14,95,96]. Both Cd and Hg also negatively affect the immune, cardiovascular, and reproductive systems [17,97]. The International Agency for Research on Cancer has classified Cd and Cd compounds as directly carcinogenic to humans in Group 1, while inorganic mercury has been evaluated as Group 3 (Not classifiable as to its carcinogenicity to humans), and methylmercury as Group 2B (Possibly carcinogenic to humans) [83].

### 3.4. Non-Carcinogenic Risk Assessment

In this study, PTM exposure levels of different milk and dairy product groups were evaluated via THQ (Table 5).

Children’s milk stands out as the riskiest group among all products in terms of THQ values. According to the mean THQ values in this product, PTMs are listed as Co > As > Cr > Cu > Al > Mn = Ni > Cd > Hg = Pb. When the THQ values for yogurt and ayran are examined, the highest mean values belong to As (0.23 and 0.16, respectively), and the effect of other PTMs is minimal. In protein milk samples, the highest mean THQ values belong to Co and As (0.07 and 0.06, respectively). The mean THQ values for all PTMs in milk and kefir were found to be close to zero. Considering that children’s milk and other dairy products other than protein milk are consumed together for the general population over the age of >15, the mean THQ values calculated for each PTM are in the range of <0.00–0.40, and the highest mean THQ value belongs to As. When all data are evaluated together, it is understood that the mean and maximum THQ values of each PTM in all products included in the study are lower than 1, and therefore, there is no non-carcinogenic health risk originating from a single PTM. Only in one sample from children’s milk products, the maximum THQ value for Co was calculated as 3.63, which is higher than the reference value. Apart from this, the maximum THQ values calculated for Cr (0.65) in children’s milk samples and for As (0.58 and 0.50, respectively) in yogurt and ayran samples are noteworthy.

**Figure 3:**
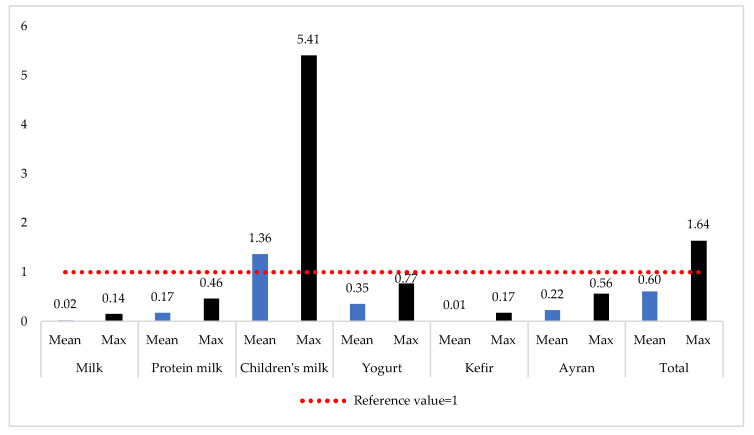
HI levels of milk and dairy products.

Within the scope of the study, PTMs’ compound exposure levels were also evaluated via HI (Figure 3).

Accordingly, since the mean and maximum HI values calculated for milk, protein milk, yogurt, kefir, and ayran are less than 1, the findings were evaluated as reliable in terms of non-carcinogenic health risks. The increase in the maximum HI value of 0.77 in yogurt samples indicates that attention should be paid, especially in high consumption scenarios. Both the mean and maximum HI values for children’s milk samples are higher than the reference value. Although the average HI value for milk, yogurt, kefir, and ayran was less than 1, the maximum HI value exceeded the reference value. Therefore, the findings should be considered as a risk signal that requires precautions to be taken against PTM contamination in milk and dairy products.

The higher THQ and HI values calculated depending on PTMs originating from milk and dairy products consumption in children compared to adults are consistent with the literature. In a study conducted in China, the mean (max) HI value for exposure of children under ≤3 years of age to 10 different PTMs originating from milk consumption was calculated as 2.42 (5.14). In the same study, the mean (max) HI values for individuals aged 3–≤14, 14–≤20, 20–≤40, 40–≤60 and >60 years of age were reported as 0.31 (0.75), 0.11 (0.27), 0.07 (0.21), 0.07 (0.18) and 0.06 (0.18), respectively [51]. In a study conducted in Romania, HI values for Cd, Cu, and Pb exposure resulting from milk consumption of adults (0.54, 14.1, and 5.38, respectively) were found to be higher than those in this study [45]. In a study conducted in Egypt, it was reported that THQ values for Ni and As exposure resulting from the consumption of toddlers’ flavored milk and dairy products (1.19 and 1.87, respectively) and HI values for all PTMs exceeded the reference value [98]. In the current study conducted in Pakistan, the HI values for Cd, Cr and Pb exposure resulting from milk/yoghurt consumption in adults were calculated as 0.09/0.03, 0.18/0.08 and 0.21/0.07, respectively, while the HI values for the same metals in children were calculated as 0.72/0.23, 1.42/0.56 and 1.61/0.47, respectively [30].

THQ and HI values show significant differences between studies. THQ and HI values are directly related to the amount of product consumption and the PTM load in the product. The amount of product consumption varies significantly according to age, gender, culture, and country. PTM load in the product also varies according to the type and variety of raw materials, production methods, and countries. This variability is thought to be the main reason for the difference between studies.

### 3.5. PTMs Contamination Level

CF values were calculated for each PTM detected in all samples included in the study. Accordingly, CF values in all samples were determined in the range of 0–762 for Al, 0–529 for Cr, 0.09–88 for Mn, 0.08–179 for Co, 0–2930 for Ni, 0.01–175 for Cu, 0–88 for As, 0–659 for Cd, 0–13.9 for Hg and 0–63 for Pb. The mean CF values calculated for each PTM detected in all samples were calculated as 101 ± 94 for Al, 76 ± 71 for Cr, 14 ± 13 for Mn, 13 ± 11 for Co, 324 ± 225 for Ni, 29 ± 27 for Cu, 12 ± 11 for As, 44 ± 56 for Cd, 2 ± 1 for Hg and 5 ± 6 for Pb. Accordingly, in all examined samples, Al, Cr, Mn, Co, Ni, Cu, As, and Cd caused pollution at the level of “very high contamination”, Pb at the level of “considerable contamination”, and Hg at the level of “moderate contamination”. The contamination levels of PTMs, as determined by the mean CF value calculated for each PTM detected in each sample, are shown in Table 6.

According to Table 6, 4 PTMs for milk, 8 PTMs for protein milk, 7 PTMs for children’s milk, 8 PTMs for yogurt, 3 PTMs for kefir, and 8 PTMs for ayran caused pollution at very high contamination levels. PTMs with low contamination levels were determined as Hg in protein milk samples, Hg and Pb in children’s milk samples, and Cd and Pb in kefir samples.

In addition, the PLI values and contamination levels of each product group examined were analyzed within the scope of the study (Table 6). The average PLI values of milk, protein milk, children’s milk, yogurt, kefir, and ayran were calculated as 3.60 ± 1.40, 15 ± 23, 24 ± 23, 23 ± 16, 3.80 ± 2.60, and 10.5 ± 19.5, respectively. PLI values for all examined product groups indicate progressive contamination.

## 4. Conclusions

This study has comprehensively revealed the PTMs levels in milk and dairy products offered for consumption in Türkiye, the possible risks they pose to human health, and the PTM contamination level. Among the six product groups analyzed, especially children’s milk and yoghurt have the highest values in terms of total PTM load. Although some PTMs, such as Hg, Pb, and Cd, are present at low levels in most samples, the levels of Al, Cr, Ni, Co, and Cu are particularly noteworthy. Although the risk level was found to be low for most products as a result of EDI, THQ, and HI calculations, it was observed that HI values in children’s milk exceeded the threshold value. It should also be noted that exposure and health risks are much higher when it is taken into account that children may consume other dairy products such as yoghurt and kefir. The significant positive correlations detected between PTMs indicate common contamination sources for these PTMs and show that contamination control should be addressed with a holistic approach. In addition, it was determined that PTMs reached very high contamination levels and PLI values, indicating advanced spoilage in some samples. In line with these findings, it is of great importance to pay attention to dairy products, especially those intended for children, to take measures to minimize the transfer of PTMs to the product, and to regularly monitor PTMs. Due to the limited number of samples used in this study, generalizations at the national level are not warranted. However, the findings may serve as an indication that more consistent and representative results could be obtained through a larger-scale study conducted over an extended period. To minimize the presence of PTMs in milk and dairy products, cleaning and sanitation practices should be rigorously applied, and equipment used in production should be made exclusively of food-grade stainless steel. Additionally, regular maintenance and monitoring of processing environments are essential to prevent contamination. By developing preventive strategies to reduce contamination risks at every stage of the dairy products supply chain, both the protection of public health and the sustainability of food safety can be ensured.

## Figures and Tables

**Table 1:** Health risk assessment formula, variables, and criteria.

Cutoff Point	Formula	Variables	Criteria
Estimated Daily Intake (EDI) (µg/kg/day)	$\mathrm{EDI}=\frac{C\;\times \;\mathrm{IR}}{\mathrm{bw}\;\times \;1000}$	C is the concentration of each PTM in each sample (μg/L–kg), IR is the intake rate of each sample (g/day), bw is the body weight (kg), and 1000 is the conversion factor. Milk and yogurt consumption of the general population (>15 years old) is 35 mL and 113 g, respectively, and body weight is 70 kg [34]. The body weight of children aged 3–6 is 17.5 kg [35]. ^a^ The consumption amount of protein milk, children’s milk, kefir, and ayran was accepted as 500, 200, 250 and 200 mL, respectively.	PTM exposure levels should not exceed the Tolerable Daily Intake (TDI) or the Recommended Dietary Allowance (RDA).
Target Hazard Quotient (THQ) (It has no units)	$\mathrm{THQ}=\;\frac{\mathrm{EDI}}{\mathrm{RfD}}$	RfD is the oral reference dose (μg/bw/day). RfD for Cr, Mn, Al, Co, Ni, Cu, As, Cd, Hg, and Pb is 3, 140, 143, 0.3, 20, 40, 0.3, 1, 0.1, and 4 μg/kg/day, respectively [36,37].	While THQ indicates a significant health problem that is not carcinogenic, THQ < 1 means that there is an insignificant risk of health hazard [38].
Hazard Index (HI) (It has no units)	$\mathrm{HI}=\sum_{i=1}^{10}{\mathrm{THQ}}_{1}$	_____	While HI ≥ 1 indicates a significant health problem that is not carcinogenic, HI < 1 means that there is an insignificant risk of health hazard [38].

^a^ The analyzed children’s milk, protein milk, kefir, and ayran products are marketed as single-use products. Therefore, the consumption amount was accepted as the amount defined on the packaging.

**Table 2:** The contamination levels for CF and PLI of PTMs [40].

CF	Contamination Level	Color Assigned to the Contamination Level
CF < 1	low contamination	
1 ≤ CF < 3	moderate contamination	
3 ≤ CF < 6	considerable contamination	
CF ≥ 6	very high contamination	
PLI		
PLI < 1	no pollution	
PLI = 1	baseline contamination level	
PLI > 1	progressive degradation	

CF: Contamination factor, PLI: Pollution load index.

**Table 3:** The mean ± SD (µg/L−kg*) PTMs level detected in milk and dairy products and comparison with other studies.

Countries	Products	Al	Cr	Mn	Co	Ni	Cu	As	Cd	Hg	Pb	References
		Mean ± SD	Mean ± SD	Mean ± SD	Mean ± SD	Mean ± SD	Mean ± SD	Mean ± SD	Mean ± SD	Mean ± SD	Mean ± SD	
Türkiye	Milk	281 ± 644	5 ± 11	16 ± 5	2 ± 1	12 ± 13	19 ± 8	8 ± 12	<LOD	0.01 ± 0.02	0.03 ± 0.08	This study
	Protein milk	424 ± 495	15 ± 22	122 ± 195	7 ± 4	40 ± 56	99 ± 27	6 ± 7	0.11 ± 0.27	<LOD	0.04 ± 0.09	
	Children’s milk	895 ± 1145	58 ± 58	300 ± 328	18 ± 26	88 ± 78	256 ± 17	6 ± 3	0.10 ± 0.15	<LOD	<LOD	
	Yogurt *	946 ± 815	56 ± 41	155 ± 149	10 ± 10	72 ± 100	183 ± 108	42 ± 37	0.02 ± 0.07	0.02 ± 0.03	0.01 ± 0.02	
	Kefir	32 ± 57	9 ± 9	29 ± 16	2 ± 1	14 ± 18	19 ± 7	4 ± 2	<LOD	0.02 ± 0.02	<LOD	
	Ayran	30 ± 40	11 ± 19	55 ± 97	3 ± 4	29 ± 43	53 ± 81	17 ± 23	0.01 ± 0.02	0.02 ± 0.03	0.09 ± 0.15	
Pakistan	Milk	___^a^	22–473	___^a^	___^a^	___^a^	___^a^	___^a^	7–69	___^a^	<LOD–608	[30]
	Yogurt	___^a^	48–441	___^a^	___^a^	___^a^	___^a^	___^a^	15–59	___^a^	133–465	
Lebanon	Yogurt	___^a^	___^a^	___^a^	___^a^	___^a^	___^a^	___^a^	6.75	___^a^	13.7	[41]
Libya	Ayran	___^a^	___^a^	___^a^	___^a^	___^a^	129 ± 57.0	___^a^	7.00 ± 1.00	___^a^	88.0 ± 41.0	[42]
Armenia	Milk	___^a^	___^a^	___^a^	___^a^	___^a^	226 ± 0.00	___^a^	2.17 ± 0.00	<LOD	0.79 ± 0.00	[43]
Brazil	Milk	___^a^	___^a^	___^a^	1050 ± 840	150 ± 310	200 ± 151	___^a^	___^a^	6800 ± 3220	3550 ± 3310	[44]
Romania	Milk	___^a^	___^a^	___^a^	___^a^	___^a^	2400 ± 62	___^a^	3.90 ± 1.10	___^a^	120 ± 44	[45]
Poland	Milk	___^a^	___^a^	___^a^	___^a^	___^a^	360 ± 110	___^a^	<0.00 ± 0.00	___^a^	12.0 ± 4.00	[46]
	Kefir	___^a^	___^a^	___^a^	___^a^	___^a^	1250 ± 220	___^a^	<0.00 ± 0.00	___^a^	156 ± 22.0	
France	Milk	69.3 ± 23.7	10.7 ± 1.15	___^a^	1.23 ± 0.68	25.0 ± 0.00	___^a^	1.00 ± 0.00	0.30 ± 0.00	___^a^	___^a^	[47]
Slovak Republic	Yogurt	___^a^	120	130	___^a^	200	10	___^a^	<0.00	<0.00	110	[48]
Iran	Milk	___^a^	___^a^	___^a^	___^a^	___^a^	378 ± 159	___^a^	1.00 ± 0.49	___^a^	9.59 ± 1.99	[49]
	Yogurt	___^a^	___^a^	___^a^	___^a^	___^a^	399 ± 125	___^a^	0.99 ± 0.40	___^a^	7.54 ± 1.76	
Spain	Yogurt	720 ± 570	20 ± 10	20 ± 4	2 ± 1	10 ± 3	290 ± 80	___^a^	<0.00	___^a^	3 ± 3	[50]
China	Milk	173 ± 92.0	10.6 ± 3.30	___^a^	3.80 ± 0.07	55.2 ± 81.3	439 ± 15.0	___^a^	1.90 ± 1.10	___^a^	6.20 ± 2.20	[51]
Republic of Korea	Milk	___^a^	365 ± 0.41	134 ± 0.47	5.71 ± 0.02	153 ± 0.26	383 ± 0.50	1.90 ± 0.07	2.38 ± 0.02	___^a^	3.35 ± 0.08	[52]
	Yogurt	___^a^	204 ± 0.32	79.3 ± 0.132	3.74 ± 0.01	87.6 ± 0.15	138 ± 0.26	0.26 ± 0.06	1.77 ± 0.02	___^a^	4.21 ± 0.07	

^a^ Not defined. * Reported as µg/kg.

**Table 4:** The mean ± SD PTM exposure from milk and dairy products consumption in the general population aged 15 > aged (µg/kg/day).

Products	Al	Cr	Mn	Co	Ni	Cu	As	Cd	Hg	Pb
	Mean ± SD (Min–Max)	Mean ± SD (Min–Max)	Mean ± SD (Min–Max)	Mean ± SD (Min–Max)	Mean ± SD (Min–Max)	Mean ± SD (Min–Max)	Mean ± SD (Min–Max)	Mean ± SD (Min–Max)	Mean ± SD (Min–Max)	Mean ± SD (Min–Max)
Milk	0.14 ± 0.32 (0.00–1.36)	<0.00 ± 0.00 (0.00–0.02)	0.01 ± 0.00 (0.01–0.01)	<0.00 ± 0.00 (0.00–0.01)	0.01 ± 0.01 (0.00–0.02)	0.01 ± 0.00 (0.00–0.02)	<0.00 ± 0.01 (0.00–0.03)	<0.00 ± 0.00 (0.00–0.00)	<0.00 ± 0.00 (0.00–0.00)	<0.00 ± 0.00 (0.00–0.00)
Protein milk	1.20 ± 1.41 (0.40–4.10)	0.04 ± 0.06 (0.00–0.20)	0.35 ± 0.56 (0.10–1.50)	0.02 ± 0.01 (0.00–0.01)	0.11 ± 0.16 (0.00–0.40)	0.28 ± 0.44 (0.10–1.20)	0.02 ± 0.02 (0.00–0.03)	<0.00 ± 0.00 (0.00–0.01)	<0.00 ± 0.00 (0.00–0.00)	<0.00 ± 0.00 (0.00–0.00)
Children milk (3–7 years old)	10.2 ± 13.1 (0.00–37.5)	0.59 ± 0.69 (0.00–1.95)	3.37 ± 3.80 (0.04–8.30)	0.20 ± 0.30 (0.00–1.09)	0.86 ± 0.88 (0.00–2.06)	2.89 ± 2.96 (0.02–7.41)	0.07 ± 0.04 (0.00–0.10)	<0.00 ± 0.00 (0.00–0.01)	<0.00 ± 0.00 (0.00–0.00)	<0.00 ± 0.00 (0.00–0.00)
Yogurt	1.53 ± 1.31 (0.00–4.08)	0.09 ± 0.07 (0.01–0.12)	0.25 ± 0.24 (0.05–0.31)	0.02 ± 0.02 (0.00–0.03)	0.12 ± 0.16 (0.00–0.13)	0.30 ± 0.18 (0.09–0.45)	0.07 ± 0.06 (0.00–0.17)	<0.00 ± 0.00 (0.00–0.01)	<0.00 ± 0.00 (0.00–0.00)	<0.00 ± 0.00 (0.00–0.00)
Kefir	0.11 ± 0.18 (0.00–0.04)	<0.00 ± 0.00 (0.00–0.09)	<0.00 ± 0.00 (0.00–0.97)	<0.00 ± 0.00 (0.00–0.02)	<0.00 ± 0.00 (0.00–0.16)	<0.00 ± 0.00 (0.00–0.66)	<0.00 ± 0.00 (0.00–0.01)	<0.00 ± 0.00 (0.00–0.00)	<0.00 ± 0.00 (0.00–0.00)	<0.00 ± 0.00 (0.00–0.00)
Ayran	0.09 ± 0.11 (0.00–0.34)	0.03 ± 0.05 (0.00–0.07)	0.16 ± 0.28 (0.00–0.25)	0.01 ± 0.01 (0.00–0.01)	0.08 ± 0.13 (0.00–0.09)	0.15 ± 0.23 (0.00–0.30)	0.05 ± 0.07 (0.00–0.15)	<0.00 ± 0.00 (0.00–0.00)	<0.00 ± 0.00 (0.00–0.00)	<0.00 ± 0.00 (0.00–0.00)
Total *	1.87 (0.00–5.82)	0.12 (0.00–0.30)	0.42 (0.06–1.54)	0.03 (0.00–0.07)	0.20 (0.00–0.40)	0.46 (0.09–1.43)	0.12 (0.00–0.36)	<0.00 (0.00–0.00)	<0.00 (0.00–0.00)	<0.00 (0.00–0.00)

* Protein milk and children’s milk exposures are not included.

**Table 5:** THQ values of PTM exposure from milk and dairy product consumption.

Products	Al	Cr	Mn	Co	Ni	Cu	As	Cd	Hg	Pb
	Mean ± SD (Min–Max)	Mean ± SD (Min–Max)	Mean ± SD (Min–Max)	Mean ± SD (Min–Max)	Mean ± SD (Min–Max)	Mean ± SD (Min–Max)	Mean ± SD (Min–Max)	Mean ± SD (Min–Max)	Mean ± SD (Min–Max)	Mean ± SD (Min–Max)
Milk	<0.00 ± 0.00 (0.00–0.01)	<0.00 ± 0.00 (0.00–0.01)	<0.00 ± 0.00 (0.00–<0.01)	<0.00 ± 0.00 (0.00–0.01)	<0.00 ± 0.00 (0.00–<0.01)	<0.00 ± 0.00 (0.00–<0.01)	0.01 ± 0.02 (0.00–0.09)	<0.00 ± 0.00 (0.00–<0.01)	<0.00 ± 0.00 (0.00–<0.01)	<0.00 ± 0.00 (0.00–<0.01)
Protein milk	0.01 ± 0.01 (0.00–0.03)	0.01 ± 0.02 (0.00–0.05)	<0.00 ± 0.00 (0.00–0.01)	0.07 ± 0.04 (0.02–0.13)	<0.00 ± 0.00 (0.00–0.01)	0.01 ± 0.02 (0.00–0.06)	0.06 ± 0.06 (0.00–0.15)	<0.00 ± 0.00 (0.00–0.02)	<0.00 ± 0.00 (0.00–<0.01)	<0.00 ± 0.00 (0.00–<0.01)
Children’s milk (3–7 years old)	0.07 ± 0.09 (0.00–0.26)	0.20 ± 0.23 (0.00–0.65)	0.02 ± 0.02 (0.00–0.06)	0.67 ± 0.99 (0.01–3.63)	0.02 ± 0.02 (0.00–0.05)	0.14 ± 0.15 (0.00–0.37)	0.22 ± 0.12 (0.00–0.34)	0.01 ± 0.01 (0.00–0.05)	<0.00 ± 0.00 (0.00–<0.01)	<0.00 ± 0.00 (0.00–<0.01)
Yogurt	0.01 ± 0.01 (0.00–0.03)	0.03 ± 0.02 (0.00–0.04)	<0.00 ± 0.00 (0.00–<0.01)	0.06 ± 0.05 (0.00–0.10)	<0.00 ± 0.00 (0.00–<0.01)	0.01 ± 0.01 (0.00–0.02)	0.23 ± 0.20 (0.00–0.58)	<0.00 ± 0.00 (0.00–<0.01)	<0.00 ± 0.00 (0.00–<0.01)	<0.00 ± 0.00 (0.00–<0.01)
Kefir	<0.00 ± 0.00 (0.00–<0.01)	<0.00 ± 0.00 (0.00–0.03)	<0.00 ± 0.00 (0.00–0.01)	<0.00 ± 0.00 (0.00–0.06)	<0.00 ± 0.00 (0.00–<0.01)	<0.00 ± 0.00 (0.00–0.03)	<0.00 ± 0.00 (0.00–0.04)	<0.00 ± 0.00 (0.00–<0.01)	<0.00 ± 0.00 (0.00–<0.01)	<0.00 ± 0.00 (0.00–<0.01)
Ayran	<0.00 ± 0.00 (0.00–<0.01)	0.01 ± 0.02 (0.00–(0.02)	<0.00 ± 0.00 (0.00–<0.01)	0.03 ± 0.04 (0.00–0.03)	<0.00 ± 0.00 (0.00–<0.01)	0.01 ± 0.01 (0.00–0.01)	0.16 ± 0.22 (0.00–0.50)	<0.00 ± 0.00 (0.00–<0.01)	<0.00 ± 0.00 (0.00–<0.01)	<0.00 ± 0.00 (0.00–<0.01)
Total *	0.01 (0.00–0.05)	0.04 (0.00–0.10)	<0.00 (0.00–0.01)	0.09 (0.00–0.20)	0.01 (0.00–0.01)	0.02 (0.00–0.06)	0.40 (0.00–1.21)	<0.00 (0.00–<0.01)	<0.00 (0.00–<0.01)	<0.00 (0.00–<0.01)

* Protein milk and children’s milk exposures are not included.

**Table 6:** The contamination levels for mean CF and PLI values of PTMs.

Products	PTMs	Color Assigned to the Contamination Level
Milk	Mn, Cd, Hg	moderate contamination
	Co, Cu, Pb	considerable contamination
	Al, Cr, Ni, As	very high contamination
Protein milk	Hg	low contamination
	As	considerable contamination
	Al, Cr, Mn, Co, Ni, Cu, Cd, Pb	very high contamination
Children’s milk	Hg, Pb	low contamination
	As	considerable contamination
	Al, Cr, Mn, Co, Ni, Cu, Cd	very high contamination
Yogurt	Hg, Pb	considerable contamination
	Al, Cr, Mn, Co, Ni, Cu, As, Cd	very high contamination
Kefir	Cd, Pb	low contamination
	Mn, Co, Cu, As, Hg	considerable contamination
	Al, Cr, Ni	very high contamination
Ayran	Hg	moderate contamination
	Pb	considerable contamination
	Al, Cr, Mn, Co, Ni, Cu, As, Cd	very high contamination
PLI		
Milk, protein milk, children’s milk, yogurt, kefir, and ayran		progressive degradation

CF: Contamination factor, PLI: Pollution load index, PTM: Potentially toxic metal.

## Data Availability

The original contributions presented in the study are included in the article/Supplementary Materials, further inquiries can be directed to the corresponding author.

## Supplementary Materials

The following supporting information can be downloaded at: https://www.mdpi.com/article/10.3390/foods14152561/s1, Table S1: Some information about the samples; Table S2: ICP MS parameters; Table S3: Analysis of the recovery, LOD, LOQ and calibration (R2) for the metals; Table S4: Concentrations (µg/L-kg*) of PTMs in milk and dairy products with statistical comparisons.
