# Supplementary material for: Occurrence of Potentially Toxic Metals Detected in Milk and Dairy Products in Türkiye: An Assessment in Terms of Human Exposure and Health Risks
Source: Foods. 2025 Jul 22;14(15):2561. doi: 10.3390/foods14152561 (PMC12346412; doi:10.3390/foods14152561)
Supplement: Supplementary file 1 [file foods-14-02561-s001.zip › foods-3768520-supplementary.pdf]

**Occurrence of Potentially Toxic Metals Detected in Milk and Dairy Products in Türkiye: An Assessment in Terms of Human Exposure and Health Risks**

Burhan Basaran [1,*]

[1] Department of Nutrition and Dietetics, Faculty of Health Sciences, Recep Tayyip Erdogan University, Rize, 53100, Türkiye.

* Corresponding Author e-mail: burhan.basaran@erdogan.edu.tr

Orcid Number of Author: 0000-0001-6506-6113

**Table S1.** Some information about the samples

| **Milk** | | | |
|---|---|---|---|
| Sample 1 | Brand A | Vitamin enriched milk | Cow's milk |
| Sample 2 | Brand B | Semi-skimmed milk | Cow's milk |
| Sample 3 | Brand A | Semi-skimmed milk | Cow's milk |
| Sample 4 | Brand A | Whole milk | Cow's milk |
| Sample 5 | Brand A | Semi-skimmed milk | Cow's milk |
| Sample 6 | Brand C | Semi-skimmed milk | Cow's milk |
| Sample 7 | Brand D | Whole milk | Cow's milk |
| Sample 8 | Brand E | Semi-skimmed milk | Cow's milk |
| Sample 9 | Brand A | Organic milk | Cow's milk |
| Sample 10 | Brand F | Organic milk | Cow's milk |
| Sample 11 | Brand G | Semi-skimmed milk | Goat milk |
| Sample 12 | Brand D | Semi-skimmed milk | Cow's milk |
| Sample 13 | Brand F | Whole milk | Cow's milk |
| Sample 14 | Brand F | Lactose free milk | Cow's milk |
| Sample 15 | Brand F | Semi-skimmed milk | Cow's milk |
| Sample 16 | Brand F | Light milk | Cow's milk |
| Sample 17 | Brand H | Whole milk | Cow's milk |
| Sample 18 | Brand H | Semi-skimmed milk | Cow's milk |
| Sample 19 | Brand I | Semi-skimmed milk | Cow's milk |

| **Protein milk** | | | |
|---|---|---|---|
| Sample 20 | Brand F | Cow's milk | Multi milk protein |
| Sample 21 | Brand F | Cow's milk | Vanilla milk protein |
| Sample 22 | Brand A | Cow's milk | Peanut milk protein |
| Sample 23 | Brand A | Cow's milk | Strawberry milk protein |
| Sample 24 | Brand B | Cow's milk | Strawberry milk protein |
| Sample 25 | Brand C | Cow's milk | Coffee milk protein |

| **Children's milk** | | | |
|---|---|---|---|
| Sample 26 | Brand H | Cow's milk | Cocoa milk |
| Sample 27 | Brand F | Cow's milk | Banana milk |
| Sample 28 | Brand J | Cow's milk | Cocoa milk |
| Sample 29 | Brand D | Cow's milk | Cocoa milk |
| Sample 30 | Brand A | Cow's milk | Strawberry milk |
| Sample 31 | Brand D | Cow's milk | Banana milk |
| Sample 32 | Brand F | Cow's milk | Cocoa milk |
| Sample 33 | Brand A | Cow's milk | Cocoa milk |
| Sample 34 | Brand A | Cow's milk | Banana milk |
| Sample 35 | Brand J | Cow's milk | Strawberry milk |
| Sample 36 | Brand F | Cow's milk | Strawberry milk |
| Sample 37 | Brand J | Cow's milk | Banana milk |
| Sample 38 | Brand K | Cow's milk | Follow-on milk |

| **Yogurt** | | | |
|---|---|---|---|
| Sample 39 | Brand A | Yogurt without cream yogurt | Cow's milk |
| Sample 40 | Brand F | Normal yogurt | Cow's milk |
| Sample 41 | Brand F | Lactose free yogurt | Cow's milk |
| Sample 42 | Brand D | Yogurt without cream yogurt | Cow's milk |
| Sample 43 | Brand R | Normal yogurt | Cow's milk |
| Sample 44 | Brand F | Organic yogurt | Cow's milk |
| Sample 45 | Brand H | Normal yogurt | Cow's milk |
| Sample 46 | Brand I | Normal yogurt | Cow's milk |
| Sample 47 | Brand B | Normal yogurt | Cow's milk |
| Sample 48 | Brand C | Normal yogurt | Cow's milk |
| Sample 49 | Brand K | Normal yogurt | Cow's milk |
| Sample 50 | Brand O | Normal yogurt | Cow's milk |
| Sample 51 | Brand S | Normal yogurt | Cow's milk |

| **Kefir** | | | |
|---|---|---|---|
| Sample 52 | Brand F | Cow's milk | Normal kefir |
| Sample 53 | Brand A | Cow's milk | Fruity kefir |
| Sample 54 | Brand A | Cow's milk | Normal kefir |
| Sample 55 | Brand F | Cow's milk | Fruity kefir |
| Sample 56 | Brand D | Cow's milk | Strawberry kefir |
| Sample 57 | Brand F | Cow's milk | Strawberry kefir |
| Sample 58 | Brand A | Cow's milk | Peach-pineapple kefir |

| **Ayran** | | | |
|---|---|---|---|
| Sample 59 | Brand A | Normal | Cow's milk |
| Sample 60 | Brand D | Normal | Cow's milk |
| Sample 61 | Brand B | Normal | Cow's milk |
| Sample 62 | Brand C | Normal | Cow's milk |
| Sample 63 | Brand F | Normal | Cow's milk |
| Sample 64 | Brand H | Normal | Cow's milk |
| Sample 65 | Brand I | Normal | Cow's milk |
| Sample 66 | Brand J | Normal | Cow's milk |

**Table S2.** ICP MS parameters.

| | Standard mode (No gas) | He mode |
|---|---|---|
| **Oxide** | 156/140 0.940 % | 156/140 0.434 % |
| **Doubly Charged** | 70/140 1.254 % | 70/140 1.049 % |
| **Plasma Parameters** | | |
| **Plasma Mode** | Low matrix | Low matrix |
| **RF Power** | 1550 W | 1550 W |
| **Rf Matching** | 1.80 V | 1.80 V |
| **Sample Depth** | 8.0 mm | 8.0 mm |
| **Nebulizer Gas** | 1.07 L/min | 1.07 L/min |
| **Nebulizer Pump** | 0.10 rps | 0.10 rps |
| **S/C Temp** | 2 °C | 2 °C |
| **Auxiliary Gas** | 0.90 L/min | 0.90 L/min |
| **Plasma Gas** | 15.0 L/min | 15.0 L/min |
| **Lens Parameters** | | |
| **Extract 1** | 0.0 V | 0.0 V |
| **Extract 2** | −200.0V | −200.0 V |
| **Omega Bias** | −70 V | −70.0 V |
| **Omega Lens** | 8.4 V | 8.4 V |
| **Cell Entrance** | −30 V | −40.0 V |
| **Cell Exit** | −50 V | −60.0 V |
| **Deflect** | 13 V | 0.0 V |
| **Plate Bias** | −35 V | −55.0 V |
| **Cell Parameters** | | |
| **He Flow** | 0.0 mL/min | 4.3 mL/min |
| **OctP Bias** | −8.0 V | −18.0 V |
| **OctP Rf** | 200 V | 200 V |
| **Energy Discrimination** | 5.0 V | 3.0 V |
| **QP Parameters** | | |
| **Mass Gain** | 147 | 147 |
| **Mass Offset** | 125 | 125 |
| **Axis Gain** | 1.0029 | 1.0029 |
| **Axis Offset** | 0.00 | 0.05 |
| **QP Bias** | −3.0 V | −15.0 V |
| **Hardware Settings** | | |
| **Torch H** | 0.4 mm | 0.4 mm |
| **Torch V** | 0.1 mm | 0.1 mm |
| **Dicriminator** | 3.9 mV | 3.9 mV |
| **Analog HV** | 2168 V | 2168 V |
| **Pulse HV** | 1065 V | 1065 V |
| **Integration Time** | 0.1 | 0.1 |

**Table S3.** Analysis of the recovery, LOD, LOQ and calibration ($R^2$) for the metals

| **Metals** | **Concentration (**μg/L) | **Mean Recovery** (%) | $R^2$ | **LOD (**μg/kg) | **LOQ (**μg/kg) |
|---|---|---|---|---|---|
| Al | 0, 10, 25, 50,100, 250, 500 | 103 | 0.9996 | 0.182 | 0.608 |
| Cr | 0, 10, 25, 50,100, 250, 500 | 110 | 0.9999 | 0.008 | 0.025 |
| Mn | 0, 10, 25, 50,100, 250, 500 | 100 | 0.9999 | 0.012 | 0.039 |
| Co | 0, 10, 25, 50,100, 250, 500 | 108 | 0.9999 | 0.008 | 0.026 |
| Ni | 0, 10, 25, 50,100, 250, 500 | 100 | 0.9998 | 0.041 | 0.138 |
| Cu | 0, 10, 25, 50,100, 250, 500 | 95 | 0.9997 | 0.038 | 0.126 |
| As | 0, 10, 25, 50,100, 250, 500 | 101 | 1.0000 | 0.007 | 0.022 |
| Cd | 0, 10, 25, 50,100, 250, 500 | 85 | 0.9998 | 0.005 | 0.016 |
| Hg | 0, 2.5, 5, 7.5, 10, 12.5 | 80 | 0.9981 | 0.004 | 0.013 |
| Pb | 0, 10, 25, 50,100, 250, 500 | 90 | 0.9998 | 0.023 | 0.067 |

**Table S4.** Concentrations (μg/L−kg*) of PTMs in milk and dairy products with statistical comparisons

| Products | Al | Cr | Mn | Co | Ni | Cu | As | Cd | Hg | Pb |
|---|---|---|---|---|---|---|---|---|---|---|
| | Median (Min−Max) | Median (Min−Max) | Median (Min−Max) | Median (Min−Max) | Median (Min−Max) | Median (Min−Max) | Median (Min−Max) | Median (Min−Max) | Median (Min−Max) | Median (Min−Max) |
| Milk | 8.64$^{b}$ (<LOD−2718) | 1.30$^{b}$ (<LOD−46.4) | 13.7$^{b}$ (11.2−28.5) | 2.36$^{b}$ (1.09−3.79) | 11.3$^{b}$ (<LOD−49.5) | 19.9$^{b}$ (4.25−33.4) | 4.52$^{b}$ (<LOD−52.8) | 0$^{b}$ (<LOD) | 0$^{a}$ (<LOD −0.10) | 0$^{a}$ (<LOD−0.35) |
| Protein milk | 273$^{ab}$ (124−1419 | 7.22$^{ab}$ (<LOD−57.4) | 30.8$^{ab}$ (24.2−514) | 6.27$^{a}$ (2.40−13.2) | 11.6$^{b}$ (<LOD−135) | 36.7$^{ab}$ (26.8−416) | 5.08$^{ab}$ (<LOD−16.2) | 0$^{a}$ (<LOD−0.66) | 0$^{a}$ (<LOD) | 0$^{a}$ (<LOD−0.22) |
| Children's milk | 265$^{ab}$ (<LOD−3277) | 58$^{a}$ (<LOD−171) | 70$^{a}$ (5.41−725) | 11.4$^{a}$ (2.05−95.2) | 105$^{a}$ (<LOD−180) | 165$^{a}$ (16.8−648) | 6.82$^{ab}$ (<LOD−9.01) | 0$^{a}$ (<LOD−0.44) | 0$^{a}$ (<LOD −0.10) | 0$^{a}$ (<LOD−0.20) |
| Yogurt* | 773$^{a}$ (<LOD−2530) | 53.8$^{a}$ (7.13−168) | 111$^{a}$ (34.7−601) | 6.07$^{a}$ (0.21−35.2) | 60.1$^{a}$ (<LOD−367) | 148$^{a}$ (32.2−409) | 38.4$^{a}$ (<LOD−108) | 0$^{b}$ <LOD−0.24) | 0$^{a}$ (<LOD−0.10) | 0$^{a}$ (<LOD−0.08) |
| Kefir | 11.7$^{b}$ (<LOD−160) | 6.39$^{ab}$ (<LOD−25.7) | 26.5$^{ab}$ (17.3−61.1) | 1.98$^{b}$ (0.96−3.00) | 0$^{c}$ (<LOD−43.0) | 20.1$^{b}$ (3.15−24.2) | 4.97$^{ab}$ (<LOD−7.36) | 0$^{b}$ (<LOD) | 0.007$^{a}$ (<LOD−0.05) | 0$^{a}$ (<LOD) |
| Ayran | 17.2$^{ab}$ (<LOD−120) | 1.98$^{b}$ (<LOD−53.8) | 12.8$^{b}$ (7.34−286) | 1.73$^{b}$ (0.86−14.1) | 15.3$^{b}$ (<LOD−132) | 13.2$^{b}$ (4.25−236) | 5.84$^{ab}$ (<LOD−55.3) | 0$^{b}$ (<LOD−0.05) | 0$^{a}$ (<LOD−0.10) | 0$^{a}$ (<LOD−0.35) |
